# Similarities and differences in key diagnosis, treatment, and management approaches for PAH deficiency in the United States and Europe

**DOI:** 10.1186/s13023-020-01541-2

**Published:** 2020-09-25

**Authors:** Tracy Brock Lowe, Jane DeLuca, Georgianne L. Arnold

**Affiliations:** 1 Clemson University, Clemson, SC USA; 2 Medical Genetics Clinical Research, UPMC Children’s Hospital of Pittsburgh, Pittsburgh, PA USA

**Keywords:** Phenylalanine hydroxylase deficiency, Phenylketonuria, Clinical guidelines, Management, Treatment recommendations

## Abstract

**Background:**

Individuals with phenylalanine hydroxylase (PAH) deficiency lack an enzyme needed to metabolize the amino acid, phenylalanine. This leads to an increase of phenylalanine in the blood, which is associated with changes in cognitive and psychological functioning. Skilled clinical management is essential for preventing complications and providing comprehensive care to patients. In the last decade, the American College of Genetics and Genomics (ACMG) and a group of European experts developed separate guidelines to provide recommendations for the management and care of persons with PAH deficiency. The purpose of this paper was to compare and contrast these guidelines in order to understand the different approaches to PAH deficiency care.

**Methods:**

We examined the procedures used to develop both guidelines, then evaluated key areas in PAH deficiency care which included screening, diagnostic approaches, dietary treatment (initiation and duration), ongoing phenylalanine level/ nutritional monitoring, neurocognitive screening, adherence issues in treatment, and special populations (women and maternal PKU, late or untreated PAH deficiency, and transitioning to adult services). We conducted a scoping review of four key topics in PAH deficiency care to explore recent research studies performed since the publication of the guidelines.

**Results:**

The ACMG and European expert group identified limited numbers of high quality studies to use as evidence for their recommendations. The ACMG and European guidelines had many similarities in their respective approaches PAH deficiency care and recommendations for the diagnosis, treatment, and management for persons with PAH deficiency. There were also a number of differences between the guidelines regarding the upper range for phenylalanine levels in adolescents and adults, the types of instruments used and frequency of neuropsychiatric examinations, and monitoring of bone health. Treatment adherence can be associated with a number of challenges, such as aversions to medical foods and formulas, as well as factors related to educational, social, and psychosocial issues. From the scoping review, there were many new studies addressing issues in treatment and management including new research on sapropterin adherence and increased dietary protein tolerance and pegvaliase on the reduction in phenylalanine levels and hypersensitivity reactions.

**Conclusions:**

In the last decade, ACMG and European experts developed comprehensive guidelines for the clinical management of phenylalanine hydroxylase deficiency. The guidelines offered background and recommendations for clinical care of patients with PAH deficiency throughout the lifespan. New research evidence is available and updates to guidelines can keep pace with new developments. Evidence-based guidelines for diagnosis and treatment are important for providing expert care to patients.

## Introduction

Phenylalanine hydroxylase (PAH) deficiency, also known as phenylketonuria (PKU), is a rare, autosomal recessive inborn error of metabolism caused by a variant of the *PAH* gene [1]. Norwegian physician and biochemist Asbjørn Følling identified PAH deficiency in 1934 [2]. Individuals with PAH deficiency lack the enzyme phenylalanine hydroxylase needed to metabolize the amino acid, phenylalanine. When this enzyme is deficient, a buildup of toxic phenylalanine metabolites occurs in the blood and brain [1]. A diet low in phenylalanine was developed in the 1950s to decrease elevated blood phenylalanine levels. Newborn screening was begun in the 1960s for early identification and instituting treatment for PAH deficiency.

Poor clinical management of PAH deficiency can result in serious neurocognitive deficits, mental health issues, and behavioral concerns for patients. Expert clinical management is essential for preventing complications and providing comprehensive care to patients. Clinical guidelines can be instrumental in providing recommendations to healthcare providers for the medical management and support of patients.

In the United States, the American College of Medical Genetics and Genomics (ACMG) developed guidelines for healthcare providers for the diagnosis, treatment, and management of patients with PAH deficiency [3]. The Genetic Metabolic Dietitians International (GMDI) and regional and national groups developed recommendations to accompany the ACMG guidelines for dietary management and ongoing assessment [4]. In Europe, a list of guidelines was compiled by experts and specialists in PAH deficiency care appointed by the European Society for Phenylketonuria and Allied Disorders Treated as Phenylketonuria (ESPKU), a parent-founded group of EU countries. The European guideline publications consist of two documents: the larger complete article and the shorter version that highlighted key points on phenylalanine diagnosis and management [5, 6]. Both the ACMG and European experts developed their guidelines through consensus and reviews of the available evidence from scientific, neurocognitive, psychosocial and treatment research. Although the respective guidelines demonstrate areas of agreement, there are divergences related to PAH deficiency treatment and monitoring. As clinicians can attest, medical management of persons with PAH deficiency is very complex. It is instructive to learn about the different approaches and recommendations from the respective guidelines in caring for persons with PAH deficiency. The purpose of this paper is to compare and contrast the guidelines and the conclusions presented by the ACMG and the priority recommendations from European experts for the diagnosis, management, and treatment of PAH deficiency. A scoping review was conducted, as part of this paper, to highlight research that was issued in select topic areas since the publication of the guidelines.

## Methods for developing PAH deficiency guidelines

The ACMG and European guidelines were developed by the ACMG working group and ESPKU-appointed committees, respectively. The ACMG is an organization of genetics professionals from a variety of backgrounds and genetic specialties. The ESPKU is multinational parent-established organization with the goal of improving treatment of phenylketonuria. A board and scientific advisory committee governs it.

The ACMG guideline-working group consisted of self- referred individuals who registered potential conflicts of interest with the ACMG. Working group members met via teleconference and in person for guideline development. There were two review processes based on the first NIH consensus conference in 2000 [3]. The ACMG relied on the National Institutes of Health (NIH) and the Agency for Healthcare Research and Quality (AHRQ) and previous NIH and AHRQ consensus information for identifying defined areas of focus for PAH deficiency management. Questions for the review were updated. Pertinent papers published before 2011 were examined with the most recent AHRQ review (3/2012) through September 2012 (workgroup meeting date). Eighty additional articles of new and more recent research were included. Each component of the guidelines was reviewed and approved by the working group. Final recommendations consisted of 75% of the participants in agreement [3]. The Scottish Intercollegiate Guideline Network (SIGN) summarized the evidence; however, most evidence scored an equivalent of C or D, which limited its use in the guideline decision–making process. The Genetic Metabolic Dietician’s International (GMDI) and other groups developed a companion document to the ACMG guidelines consisting of nutritional and other clinical recommendations. The GMDI developed these guidelines using peer-reviewed and gray literature plus consensus methods such as the Delphi process, expert panels, and surveys [4].

For the European expert review, a panel of PAH deficiency experts was assembled by the scientific advisory committee of the ESPKU. Working groups consisted of a mix of pediatric and adult metabolic physicians, neurologists, dieticians and psychologists. The expert group developed the comprehensive consensus guidelines for PAH deficiency diagnosis and management [5]. Treatment of PAH deficiency in Europe can vary from nation to nation and guideline development was considered crucial for creating consistent care across countries. Support for a project manager was provided by ESPKU, but no other influence extended to guideline development. Five different breakout groups formed to address different aspects of PAH deficiency care:

1. Nutritional Treatment and Biochemical and Nutritional follow up
2. Psychological Outcome and Adherence
3. Neurocognitive Outcomes including Imaging
4. Adult and maternal PKU; Late Diagnosed and Untreated PKU
5. Diagnosis and Treatment initiation; Pharmacological Treatment

All literature published before Dec. 31, 2015 was included in the review. The groups reviewed the literature and determined the level of evidence using the SIGN method. A grade of A or B was acceptable. Evidence lower than a B grade was assessed using the Delphi method. Seventy recommendations were formulated using the Appraisal of Guidelines for Research and Evaluation (AGREE) method [5]. European experts identified ten key guidelines, based on the original 70, deemed most significant in PAH deficiency management [6]. The authors concluded that high quality studies were difficult to find, and study participant numbers were often small. Evidence was particularly sparse in areas of early treatment and adult management. While the shortcomings in the statistical power of the studies was acknowledged, the compilation of the key points of the literature still provided important information for PAH deficiency care. The European experts opined that guidelines might change with new evidence. A thoughtful commentary by Vockley, Chapman, and Arnold is available on the challenges of guideline development and conducting clinical research in small rare disease samples [7]. They suggest that clinical guidelines serve as a starting point for further discussions; guidelines should invite comment and allow for ongoing updating and renewal.

Supplement 1 is an index of topic headings and subheadings in the ACMG, comprehensive European guidelines and the shortened version of the guidelines. This provides a quick reference to locate each topic and their corresponding page numbers in the published articles.

## Screening and diagnosis

ACMG and European expert guidelines recommend screening all newborns for PAH deficiency [3, 5]. Newborn screening allows for the early identification and prompt initiation of treatment for PAH deficiency. In the United States, newborn screening is not one federally monitored program. All 50 states have newborn screening programs and include PAH deficiency as part of their screening panels [8]. Newborn screening in Europe varies among countries and not every nation offered routine NBS screening for PAH deficiency [9]. European experts agree that newborn screening is a “national obligation” (p.5) [5]. They cite challenges to universal screening which include variations in nations’ economic stability and the movement of immigrants between countries [5].

Both guidelines recommend that newborn screening for PAH deficiency occur within the first days of life. The ACMG suggests phenylalanine levels can be quantified within 24 h [3], but newborn screening samples are obtained typically around 24–48 h of life [10]. According to the European guidelines, newborn screening samples are obtained at 24–72 h of life [5].

The types of diagnostic testing from the ACMG and European recommendations for infants with a positive newborn screen for elevated phenylalanine are in Table 1. European experts recommend the diagnosis of PAH deficiency based on the highest serum phenylalanine level. From the European guidelines, phenylalanine: tyrosine ratio testing is not supported because it does not appear to provide clear clinical benefit [5]. Key European guidelines also recommend additional diagnostic testing to rule out potential liver disease.

**Table 1:** Testing Recommendations at Time of Diagnosis

	ACMG 2014	EU Guidelines (2017)
**Establishing Diagnosis**	Plasma amino acid analysis	Highest NBS phe level
	Phenylalanine/tyrosine ratio	Evaluate for liver disease
**BH4 testing for disorders of tetrahydrobiopterin metabolism**	Recommended with elevated Phe levels	Recommended with elevated Phe levels and/or neurological findings
	Blood/ urine tests for potential BH4 deficiency diagnoses, if abnormal, reflex to enzyme (ACMG) or rapid sequencing European guidelines	
**Genotyping**	Recommended	Not specified, but may be useful in determining BH4 responsiveness

Tetrahydrobiopterin (BH_4_) deficiency disorders are associated in some cases with elevations in serum phenylalanine levels due to a lack of tetrahydrobiopterin, a cofactor needed for PAH activity. ACMG and European experts recommend BH_4_ testing for all individuals with elevated serum phenylalanine [3, 5]. If a BH_4_ deficiency disorder is suspected, additional blood and urine testing is obtained to measure pterins [3].

The ACMG recommends genotyping for all patients diagnosed with hyperphenylalanemia [3]. Genotyping may predict disease severity and treatment response [11] in some cases. In contrast, European experts do not specify genotyping as a front line test, but suggest that it may be helpful in determining BH_4_ responsiveness or diagnosing protein dysfunction [4, 5].

## Treatment initiation and duration

Initiating treatment early in life is important for preventing neurological complications [3, 5]. According to ACMG recommendations, treatment should be started within one week of age [3], similarly, European experts recommend treatment initiation before the first ten days of life [5]. Treatment for infants requires the use of phenylalanine free metabolic infant formula in combination with breastmilk and/or infant formula [12].

Treatment initiation is based on the blood phenylalanine level. The goal of early treatment is to lower plasma phenylalanine levels quickly to the desired treatment range of 120–360 μmol/L [3, 4]. Neither the ACMG nor the European group recommend treatment if untreated blood phenylalanine levels are less than 360 μmol/L [3, 5] (Table 2). The ACMG recommends lifelong treatment of all individuals with untreated blood phenylalanine levels greater than 360 μmol/L. They cite evidence from studies indicating that milder elevations of phenylalanine, in the 360–600 μmol/L range, may lead to impaired neurocognitive functioning [3].

**Table 2:** Recommended Therapeutic Phenylalanine Treatment Ranges

	ACMG (2014)	European Guidelines (2017)
**Recommended treatment ranges**		
**Infants (1 year or less)**	120–360 μmol/L	< 360 μmol/L
**Children < 12**	120–360 μmol/L	< 360 μmol/L
**Adolescents**	120–360 μmol/L	> 600 μmol/L
**Adults > 18**	120–360 μmol/L	< 600 μmol/L
**Women**		
Prior to conception	120–360 μmol/L	< 360 μmol/L
During Pregnancy	120–360 μmol/L	120–360 μmol/L

The European guidelines differ from the ACMG by varying target phenylalanine level recommendations according to patient age, magnitude of untreated levels, and the range of phenylalanine control [5]. There is limited evidence to suggest levels could be safe for children in the 360–600 μmol/L range and the European guidelines recommend treatment for phenylalanine levels above 360 μmol/L for children to age 12. The European guidelines recommend that children with untreated phenylalanine levels between 360 and 600 μmol/L receive treatment until age 12 as long as levels subsequently remain < 600 μmol/L. For adults, lifelong treatment is recommended for phenylalanine levels greater than 600 μmol/L [5, 6] (Table 3).

**Table 3:** Recommended Duration of Treatment for PAH Deficiency

	ACMG (2014)	European Guidelines (2017)
**Recommended Duration and Treatment ranges**		
**Infants 1 year or younger**	Maintain Diet for Life Levels 120–360 μmol/L	> 360 μmol/L
**Children < 12**		> 360 μmol/L treated 360–600 μmol/L untreated
**Adolescents**		> 600 μmol/L
**Adults > 18**		> 600 μmol/L
		Maintain Diet for Life Levels > 600 μmol/L
**Women**		
Prior to conception	Maintain Diet for Life Levels 120–360 μmol/L	120–600 μmol/L (for age > 12)
During Pregnancy		120–360 μmol/L

Both the ACMG and European experts recommend periodic monitoring of infants who are untreated with levels less than 360 μmol/L to ensure these levels do not rise over time and require treatment.

## Ongoing monitoring of biochemical, nutritional, and clinical parameters

### Phenylalanine levels

Continuing monitoring of phenylalanine levels helps clinicians provide recommendations to patients and families in the management of diet and lifestyle so therapeutic phenylalanine levels can be maintained. The frequency of routine monitoring of phenylalanine levels vary according to age and level of control for the ACMG and European guidelines. Table 4 illustrates the recommendations for phenylalanine monitoring based on age group. Phenylalanine levels should be tested for all age groups during times of increased growth, if phenylalanine levels are out of treatment range, or treatment changes are initiated [3, 5].

**Table 4:** Recommendations for Routine Phenylalanine Monitoring ^a^

	ACMG (2014)	European Guidelines (2017)
**Infants**	Weekly	Weekly
**Children < 12**	Every two weeks or monthly	Fortnightly
**Adolescents**	Once monthly	Once monthly
**Adults**	Once monthly	Once monthly

^a^ Increase testing frequency recommended for out-of-treatment range phenylalanine levels

### Nutritional monitoring

The ACMG/ Nutritional management guidelines and the European experts agree that biochemical nutritional assessments should be performed routinely (Table 5). Additional monitoring is appropriate when nutritional status is in question or compromised [3–5]. The nutritional management guidelines suggest that plasm amino acids be performed frequently during infancy, monthly to every three weeks and weekly to monthly during pregnancy. Otherwise, plasma amino acids can be performed at each clinic visit [4] The ACMG suggests that plasma amino acids can also be performed when an individual has poor dietary intake and if a large portion of the diet consists of medical foods that may be nutritionally incomplete. The key European guidelines recommend annual plasma amino acid analysis [4, 5].

**Table 5:** Recommendations for Type/Timing of Routine* Nutritional Monitoring across the Lifespan

ACMG/Nutritional Management Guidelines* [3]	European Guidelines [4, 5]
**Infants (to age 1)**	**All age groups**
Plasma amino acids- *Monthly to every 3 months* Complete blood count- *Once* Albumin - *Once* Prealbumin - *Once* Ferritin -*Once* Vitamin D 25-OH- *Once*	Annual measurements of plasma amino acids, homocysteine and or methylmalonic acid (B12 markers), hemoglobin, mean corpuscular volume, and ferritin.
**Children, Adolescents, and Adults (age 1 to Adulthood)**	
Plasma amino acids- *Each clinic visit* Complete blood count- *Yearly* Albumin- *Six to 12 months* Prealbumin- *Six to 12 months* Ferritin- *Yearly* Vitamin D 25-OH- *Yearly*	

*Micronutrient or other nutritional testing can be performed due to growth concerns, poor nutritional status, or inadequate adherence to diet: zinc, copper, B12, B6, erythrocyte folate, Vitamin A, selenium, essential fatty acids, and comprehensive metabolic panel [x]

*Vitamin and mineral micronutrient testing can be performed if clinically indicated, to include calcium, zinc, selenium, and parathyroid hormones

### Neurocognitive screening

Untreated PAH deficiency can lead to intellectual disabilities. Elevated phenylalanine levels are associated with changes in neurocognition. Elevated phenylalanine levels are associated with increased rates of anxiety [13], depression [14], and attention deficit hyperactivity disorder [15]. Neurocognitive and mental health assessments are important and recommended as a routine component of care for individuals with PAH deficiency. The ACMG provides a list of screening tools and guidelines for the age and frequency of psychological and neurocognitive assessments (page 10) [3]. These are based on consensus recommendations from a panel of psychologists and include tests for executive functioning, behavior/emotion, depression, intellectual development, and adaptive skills. Neurocognitive screening is suggested every two to three years and as indicated in individuals of all ages. The key European recommendations call for frequencies of neurocognitive screening that differ from the ACMG guidelines. Psychological screening per the European guidelines is conducted for children at ages 12 and 18. Choices of neurocognitive tests can differ, but may include visuospatial functioning, executive functioning, working memory, and motor control. A table of research studies for psychological outcomes and phenylalanine levels is provided on pages 6–7 of the key guidelines document [6]. Administration of quality of life measures and psychosocial functioning are also suggested. Ensuring that appropriate screenings are performed is an important part of care. These can lead to referrals for educational or psychological services if needed and improving patient outcomes [3, 5].

### Bone health monitoring and treatment

There were differences between the guidelines recommendations for assessment of bone density and bone health. The ACMG states there is insufficient evidence to establish the need for routine bone density scans, however, the nutritional management recommendations suggest radiological bone scans begin in childhood and continue into adulthood [3, 4]. The nutritional management guidelines suggest that patients with multiple fractures or low vitamin D levels may warrant a bone density scan. European experts recommend initial bone density testing in late adolescence. If the results are stable for two consecutive years, further bone density testing is not necessary unless it becomes clinically indicated. Bone density is not routinely obtained in children or adults.

### Clinical follow up

The nutritional management companion document to the ACMG guidelines [4] suggests regular clinic visits for dietary assessment and anthropomorphic measurements Table 6. The key European guidelines specify different times for clinic visits according to age. Both guidelines recommend a team approached using providers specializing in metabolic clinical care. This can include a biochemical geneticist, dietician, psychologists, social workers, and others.

**Table 6:** Recommendations for Routine Clinical Visit Follow up Through the Ages^a^

ACMG/ Nutritional Management Guidelines (2014)	European Guidelines (2017)
**Infants (0–1 year)-** *Monthly*	**Infants (0–1 year)-** *Every 2 months*
**Children (1–7 years)-** *Monthly to every 6 months*	**Children/Adolescents (1–18 years)-***Twice yearly*
**(8–18 years)-** *Every 6–12 months*	
**Adults (> 18 years)-** *Every 6–12 months*	**Adults- (> 18 years)**- *Once yearly*
**Pregnancy-** *Monthly to every trimester*	**Pregnancy-** *Once per trimester*
**Postpartum/lactating-** *6 weeks postpartum then every 6 months*	

^a^ Increased visits due to circumstances or indications: adherence issue, treatment changes, new social circumstances

## Individualized treatment plans

The ACMG/ nutritional management guidelines promote the development of an individualized treatment plan to address each patient’s unique metabolic and social profile [3]. The key European guidelines recommend attention to individual patient requirements for dietary phenylalanine and protein tolerance, energy requirements for age, cultural and religious background and the consideration of quality of life effects of PKU care [5].

Both the ACMG/nutritional management guidelines and the European guidelines suggest the importance of planning for care during serious intercurrent illnesses with increased monitoring [3–5]. The European guidelines highlight the effects of illness and fever on phenylalanine control, which can lead to protein catabolism and elevations in phenylalanine levels. Appropriate use of antipyretics and adequate consumption of phenylalanine-free amino acid supplements should be maintained during illness to help prevent excess protein catabolism [6].

## Dietary treatment

Dietary treatment is the primary therapy for PAH deficiency worldwide. Both the ACMG and the key European guidelines stress the importance lifelong treatment for PAH deficiency. These groups similarly recognize that dietary treatment is trifold. It includes the elements of low protein diet, use of phenylalanine-free medical foods, and the use of modified low-protein foods. The companion nutritional recommendations developed by the Genetic Metabolic Dieticians International (GMDI), Southeast Regional Newborn Screening, and Genetics Collaborative [3, 4] are cited by the ACMG for guidance in dietary management. The European guidelines discuss maximizing natural protein intake as tolerated to reach the target blood phenylalanine goal of 120–600 μmol/L or less per age and growth recommendations [5].

Nutritional deficiencies are a concern for patients with limited phenylalanine tolerance who may rely more heavily on foods with limited natural protein and mineral content and metabolic formulas. Phenylalanine deficiency may occur due to over restriction of dietary phenylalanine, but this is rare [5]. Symptoms include anorexia, rash, and poor growth [5]. Ongoing monitoring is important to ensure good nutrition and therapeutic phenylalanine levels. Below therapeutic phenylalanine levels (< 120 μmol/L) may be satisfactory in persons with little dietary restriction [3], The European guidelines stress the importance of avoiding the artificial sweetener aspartame [5, 6].

### Low protein diet

The ACMG and European experts agree that a phenylalanine-restricted diet is the mainstay in lowering blood phenylalanine levels. Thus, individuals with PAH deficiency must limit natural proteins from their diets, such as meats, nuts, and dairy products. Protein intake should be tailored to the patient based on age, weight, and phenylalanine tolerance. Protein intake should promote optimal growth and development without causing a rise in blood phenylalanine levels [4]. Most non-starchy fruits and vegetables are naturally low in phenylalanine and not expected to affect blood phenylalanine levels [5].

### Medical foods and modified low protein products

Medical foods, including formulas and commercial low protein food products, are a part of therapy for individuals with PAH deficiency. These foods help provide necessary calories, fats, protein, and nutrients to maintain optimal health. Modified low protein foods contain lower levels of protein [3, 5]. These foods can substitute for standard foods to decrease protein intake. Low protein foods offer different types and varieties of foods to consume, which can help ensure adequate calorie intake, and assist individuals in remaining compliant with diet [3–5]. The nutritional management guidelines offer a table of examples of types of medical food products available in the U.S. (p. 126) [4]. Commercial food low protein products can be expensive, and costs may prohibit obtaining them. For some patients in the United States a private, third-party payer system is in place [16]. Medical foods may not be covered by insurance providers and can add substantial financial burdens to families [17]. There are different health organizations across European nations, with variations in coverage according to the system with variable affordability and availability of medical foods [18].

### Formulas

Medical foods include the use of phenylalanine-free amino acid formulas that provide supplemental protein and nutrients. These formulas meet the nutritional requirements for persons with PAH deficiency [3, 5]. A variety of preparations are available, including ready to drink and gel options. Since protein restriction can be severe in some individuals, formula supplementation is imperative in these instances to ensure adequate protein intake [3]. The nutritional status of individuals using medical foods should be routinely evaluated to ensure they meet metabolic needs to account for growth [3–5]. Without adequate protein intake, catabolism can occur in the body resulting in increased phenylalanine levels [4]. It can be a delicate balance to provide sufficient protein for growth and maintain therapeutic phenylalanine levels.

### Glycomacropeptide

Glycomacropeptide (GMP) is an alternative phenylalanine-free protein source. It is obtained from cheese whey and can be used to produce a large variety of low-phenylalanine foods [19]. The ACMG guidelines suggest that a variety of low protein food products increase patient choices and promote treatment compliance, however, ongoing nutritional monitoring is needed [3]. The key European guidelines referred to limited evidence, at the time of publication of the guidelines, on the safety and use of GMP [5].

### Large neutral amino acids

Large neutral amino acids (LNAA) are another treatment option for PAH deficiency. High concentration of LNAA may prevent phenylalanine uptake in the gut and block transport of phenylalanine into the brain [20]. LNAA treatment is suggested for adolescents and adults only. There was limited available information at the time on its effects on growth and development [3, 5] and research was suggested to determine the effects and safety of LNAAs [3]. Key European guidelines discussed LNAA and their effect on the pathophysiology of phenylalanine hydroxylase deficiency, and provide a discussion on using LNAA supplements in treatment and the mechanism of action of LNAA in the brain [5]. They agreed there was limited evidence to make a recommendation at that time regarding daily use.

## Adherence to treatment

Adherence to treatment for persons with PKU means performing specific, multiple tasks in daily treatment, conduct monitoring of phenylalanine levels, and clinical follow up. Per the ACMG and nutritional management guidelines, adherence to treatment reflects phenylalanine levels and medical food intake [3, 4]. Adherence is predicated on planning and organizational skills. Executive functioning may be impeded by high phenylalanine levels and patients may have difficulties performing the activities of treatment [3, 5]. Treatment adherence can be encouraged by understanding the benefits of treatment, having access to treatment, through the work of families, and the belief that persons with PAH deficiency can be successful in treatment [3, 4]. Treatment adherence diminishes with age [4, 5]. This is likely due to time demands of school, work, and family. Clinic and community based programs, camps, and support programs can help educate families and patients in PKU self-care. The further development of effective adherence strategies through research is needed [4].

In the development of the European guidelines, a working group was devoted to issues of psychosocial outcomes and adherence [5]. They suggest that avoiding judgmental attitudes and supporting patient motivation improved adherence. The guidelines cited research pertaining to the guilt and anxiety patients experience as a result of poor adherence to treatment and monitoring protocols, all of which can affect quality of life [5]. In recent years, better tasting PKU supplements and formula were introduced that might help patients adhere to treatment [5]. There is a lack of research devoted to understanding adherence issues to performing regular phenylalanine monitoring. The authors suggest that easier, at home testing methods may help with adherence [5].

## Pharmacological treatment

There are several pharmacological treatment advances available for PAH deficiency. The ACMG guidelines provide a detailed overview of these options. Key European guidelines offer a brief discussion about pharmacotherapy, while the complete European guidelines present more information about these pharmacological treatments.

### Sapropterin Dihydrochloride

The earliest and most widely used pharmacologic treatment to reduce phenylalanine levels in PAH deficiency is sapropterin dihydrochloride. This medication is a synthetic form of naturally occurring tetrahydrobiopterin, which is a co-factor needed for phenylalanine metabolism. Sapropterin is a form of chaperone drug which help to fold and stabilize the PAH protein. Both the ACMG and the European experts recommended that all individuals with PAH deficiency undergo a trial of sapropterin dihydrocholoride to determine if they are responsive to treatment. The starting dose for sapropterin dihydrochloride responsiveness testing is typically 10–20 mg/kg of body weight, with 20 mg/kg of body weight as the most common starting dose [3]. The European guidelines acknowledge the limited availability in supporting evidence at the time for use of the medication. The key difference between the guidelines is the contrast between when and how long to perform responsiveness testing for the drug.

Responsiveness to treatment with sapropterin dihydrochloride varies among individuals with PAH deficiency. Studies have shown that one-quarter to one-half of individuals with PAH deficiency are sapropterin dihydrocholoride responders [3]. The ACMG proposes that sapropterin dihydrochloride responsiveness is determined with a reduction of at least 30% in blood phenylalanine levels, improvement of neuropsychiatric symptoms, or when improvements in phenylalanine tolerance has been demonstrated [3]. According to ACMG guidelines, an improvement in any of these parameters justifies continuation of sapropterin dihydrochloride [3]. Sapropterin has been used with PAH deficiency in youngsters less than four and those with biosynthesis abnormalities [3]. European experts state that responsiveness to sapropterin is demonstrated when an individual increases their natural protein tolerance by at least 100% or phenylalanine levels are within the target range more than 75% of the time when tested. The key European guidelines suggest that this treatment should be used in individuals that have improved phenylalanine tolerance and blood phenylalanine levels [5].

There is a key difference between guidelines for the length of a sapropterin dihydrochloride trial period. The ACMG suggests that responsiveness testing is performed over a four-week period. European experts recommend a short-term sapropterin dihydrochloride loading test over 48 h. If individuals respond to the sapropterin dihydrochloride during the short-term test, then a longer trial of a few weeks or months is recommended.

The European guidelines [5] noted that no serious adverse events occurred with the use of the medication. Decreases in blood phenylalanine level were noted with sapropterin use, with no apparent deterioration in neurocognitive functioning or negative effects for quality of life.

The ACMG suggest at the time of guideline publication that sapropterin may be safe for use during pregnancy and may be considered an option for pregnant women where the fetus is at risk from high phenylalanine levels. There was discussion in the European guidelines of cases of pregnant women with PAH deficiency who had good pregnancy outcomes while taking sapropterin with no ill effects to the mother.

### Pegvaliase

Pegvaliase, known by the brand name Palynziq (also known as polyethylene-glycol conjugated phenylalanine ammonium lyase or PEG-PAL), is a newly approved (2019) subcutaneous injectable treatment for PAH deficiency. Both the ACMG and the complete European guidelines mention PEG-PAL as an emerging treatment [3, 5].

### Newer therapies

The main goal of all therapies is to decrease phenylalanine levels. Current PAH deficiency therapies are customized to help individuals meet their nutritional and biochemical goals. Ultimately, improving phenylalanine control may in turn enhance quality of life for affected individuals. Neither the ACMG nor the key European guidelines discussed additional emerging therapies in depth, but mention gene therapy, enzyme replacement, and hepatocyte transplant as potential therapies for PAH deficiency [3, 5].

### Special populations

Some individuals with PAH deficiency require specialized treatment plans. These are customized to meet specific nutritional, biochemical, and educational requirements of these groups. Special populations include women and maternal PKU, individuals who were untreated or received late treatment for PAH deficiency, and those transitioning from pediatric to adult care.

### Treatment recommendations for women and maternal PKU

Maintaining good metabolic control for women with PAH deficiency can prevent the effects of maternal PKU (MPKU), if a woman becomes pregnant. Table 2 illustrates the recommended therapeutic phenylalanine treatment ranges for women. Women of childbearing age and pregnant women with PAH deficiency require close monitoring of phenylalanine levels. Evidence suggests that phenylalanine levels greater than 360 μmol/L during pregnancy are linearly associated with increasing neurocognitive and neuropsychiatric complications in their offspring [3]. Elevated phenylalanine levels are teratogenic to a developing fetus and can lead to abnormalities such as microcephaly, poor fetal growth, and heart defects. The complete European guidelines note the toxic effects of elevated phenylalanine levels on the developing fetal brain.

The ACMG and key European guidelines assert that women of childbearing age should receive treatment prior to conception and during pregnancy, particularly if untreated blood phenylalanine levels are above 360 μmol/L. Both ACMG and European guidelines recommend maintaining blood phenylalanine levels between 120 and 360 μmol/L prior to conceiving and throughout pregnancy [3, 5]. The European guidelines suggest levels of 120–600 μmol/L for non-pregnant women older than age 12.

The two guidelines differ in management recommendations of PAH deficiency during pregnancy. The ACMG discusses the importance of good phenylalanine control, use of standard prenatal vitamins, and treatment and monitoring options. The guidelines recommend that women already taking sapropterin dihydrochloride prior to pregnancy should be offered the option to continue it during pregnancy. Screening ultrasounds are suggested to assess fetal growth and cardiac function. The ACMG guidelines stress frequent monitored if levels are between 360 and 600 μmol/L, with a focus on dietary phenylalanine requirements and overall nutrition monitoring. This may vary throughout pregnancy requiring adjustments to diet and phenylalanine intake [3].

The key European guidelines highlight the frequency of phenylalanine testing prior to and during pregnancy. The guidelines suggest women use birth control until phenylalanine levels are within therapeutic levels for at least two weeks prior to conception. Women with unplanned pregnancies should be evaluated in the clinic as soon as possible. Phenylalanine levels should be monitored at least twice weekly prior to conception to ensure that good control is maintained. The key guidelines further specify the need to monitor phenylalanine levels twice weekly during pregnancy. Like the ACMG recommendations, the key European guidelines recommend fetal ultrasound to assess fetal growth and organ development [5].

Both the ACMG and the key European guidelines encourage breastfeeding by women with PAH deficiency. If their infants do not have PAH deficiency, the infants can metabolism the extra phenylalanine in breast milk [3, 5]. Women without PAH deficiency can breastfeed an infant with PAH deficiency by alternating breast milk with phenylalanine- free infant formula [5] with frequent monitoring of phenylalanine levels in the infant.

### Late or untreated PAH deficiency

The ACMG and key European guidelines discussed issues in late and untreated PAH deficiency. Beginning treatment for persons with early untreated PAH deficiency may provide benefit by lowering phenylalanine levels. Research suggests that late-treated individuals may have neurological and psychological gains after instituting treatment. Both guidelines recommend initiating a six-month treatment trial in individuals with late-treated PAH deficiency. The ACMG suggests that individuals can discontinue treatment if no improvement is observed following the treatment trial [3]. The key European guidelines suggest treatment be considered on a case-by-case basis [5]. The complete, key European guidelines, and ACMG suggest screening individuals with intellectual impairments for PAH deficiency if they have emigrated from countries that may not have newborn screening, who are older and suspected of having untreated PAH deficiency, or are women with developmental disabilities or who have children with symptoms of MPKU.

### Transitioning to adult services

Transitioning to adult services from pediatric care is very important for the successful independent maintenance of treatments and keeping phenylalanine levels within therapeutic range. Designated metabolic services for adults may not be available, so patients are followed by pediatric metabolic and genetic services. The stresses of adulthood for persons with PAH deficiency may cause lapses in treatment. There may competing time demands, insurance issues or other factors that impede appropriate PAH deficiency care.

The ACMG recommends that education about transitioning begin in childhood and more responsibility for self-care assumed by the young person as they age [3]. Pregnancy counseling for young women is of paramount importance and should be discussed prior to adolescence with conversations continuing during the teenage years. The European experts are in agreement with early counselling for pregnancy for women with PAH deficiency. Self-care is emphasized with transitioning beginning around age 12 years [5]. A reasonable time line for transitioning can be configured, with responsibilities gradually assumed over time for testing and diet management.

## Genetic counseling

The ACMG recommends genetic counseling for persons and families affected by PAH deficiency and lifelong counseling for individuals with PAH deficiency [3]. This can consist of discussions of disease inheritance patterns, recurrence risk, or other issues germane to living with PAH deficiency. Genetic counselors can offer carrier testing for individuals with a family history of PAH deficiency. In addition, genetic counselors may recommend testing for PAH deficiency in females with a history of intellectual disability or if they have infants displaying symptoms of maternal PKU syndrome [3]. The European guidelines do not explicitly discuss genetic counseling; however, discussions of prenatal diagnoses may be dependent upon different countries’ laws and ethical considerations [5].

## New research since guideline publication

After the detailed comparison of the ACMG and European guidelines, we undertook a scoping review to examine the trends in research in PAH deficiency since the publications of the ACMG and European guidelines (2014–2019). Many researchers explored a wide range of issues in diagnosis, treatment, and outcomes during that time period. We could not address each topic in the guidelines, because of the magnitude and numbers of studies that were conducted. We limited our review to four main areas; neurocognitive functioning, quality of life, bone health, and the newer treatments; and sapropterin and pegvaliase to explore the types of research and evidence produced in the intervening years.

### Neurocognition

Untreated PAH deficiency is associated with high blood phenylalanine levels and intellectual disabilities. Poor dietary control is associated with psychiatric problems including behavioral issues, depression, anxiety, and phobias [3]. Discrete changes in neuropsychiatric findings of altered executive function and reaction times have been identified for persons with PAH deficiency [5]. Both the ACMG and European guidelines suggest early, sustained treatment to lower phenylalanine levels (with some variation in treatment cut offs), and ongoing neuropsychiatric assessments (with variations in ages for timing of screens) as a routine part of care so problems can be identified and appropriate interventions can be applied [3, 5]. Current research examined PAH deficiency outcomes and risk of neurocognition complications.

Recent studies examined the influence of phenylalanine levels on neurocognitive function, indicating that lower phenylalanine levels were associated with improved neurocognitive function [21, 22]. Evidence suggests that the 360 μmol/L upper target for phenylalanine levels may be too high and neurocognitive benefit may be observed at lower target values in some cases [23]. Early therapy initiation may preserve cognition, with attention problems and hyperactivity associated with poor treatment adherence [24]. In a study applying numerous tests and assessments in a modest number of early and consistently treated adults, lower IQ scores were appreciated compared to matched controls. These impairments included deficits in executive functioning and slower response time. Variability in skills was noted for study participants, with 25% demonstrating some elements of cognitive impairment [25]. A systematic review suggested that despite early treatment, neurocognitive deficients persisted in life, however, inconsistences across these findings might be attributed sampling variability, sensitivity of the chosen assessment tools, and other factors [26]. LNAA-containing metabolic formulas may help improve cognition and wellbeing in adults despite increased blood phenylalanine levels, but more research is needed [27].

### Quality of life

Quality of life (QoL) is an important concern of persons living with PKU, but a good quality of life may be hard to achieve. Previous research suggested a lower quality of life may be common for patients with PAH deficiency. The ACMG suggests that the impact of therapies and how they are delivered may affect QoL [3]. Psychiatric and behavioral issues can influence QoL. Reducing the difficulties in achieving diet compliance, which can in turn foster better phenylalanine control, should be a goal of therapies [5]. Diet treatment may be difficult, but returning to diet appears to improve quality of life in some patients [6]. The nutritional management guidelines suggest that age- specific programs, educational camps, and support programs promote adherence and improve QoL [4]. The European guidelines suggest that validated QoL measures can be used to monitor treatment impact over time [6].

Recent studies from China and Europe corroborate that lower quality of life may be common in persons with PAH deficiency. PAH deficiency management can negatively impact patients’ lives, with age and occupation substantively influencing QoL outcomes [28, 29]. Difficulty in adhering to treatment may affect QoL [24]. Adults with PAH deficiency reported lower health-related QoL in areas of cognition and mood, while affected children younger than age 12 on BH4 therapy reported similar QoL scores as their unaffected peers [30]. On a hopeful note, a qualitative study from Norway identified common themes for persons living with PAH deficiency that included gratitude for parental support, access to free prescription protein substitutes, and the affordability of low-protein special foods as positive factors in coping with the disorder [31]. Cazzorla et al. identified four key factors in a study of QoL in Italian patients with PAH deficiency. These included less awareness of PKU as a diagnosis that could be affected by dietary adherence, level of patient responsibility for care, family characteristics, and educational level all of which could affect patient compliance treatment and in turn patients’ lives [32]. The ACMG and European guidelines suggest that a healthy QoL is attainable for persons with PAH deficiency. Research suggests that more work is needed in the area of QoL studies to help improve patient outcomes.

### Bone health

PAH deficiency may affect bone development and health. There are different recommendations from the ACMG and European experts with regard to frequency and indications for performing dual-energy X ray absorptiometry. In recent research, lower bone alkaline phosphatase and possible bone mineral disease was observed in a cohort of patients with PAH deficiency [33]. Bone strength may be decreased due to alterations in bone metabolism and neuromuscular abnormalities [34]. Recent studies suggest that bone density may be slightly lower in individuals with PAH deficiency, though still fall within the normal limits. A precise relationship between phenylalanine levels and bone health is not apparent [35, 36]. In one study, males were found to have lower bone density scores than females, possibly related to higher intake of medical foods and calcium loss through urinary excretion. Males may be at greater risk for osteoporosis, but more research is needed for specific treatment recommendations [37]. Evidence indicates calcium supplements may be beneficial to bone health [38]. If there is risk for poor outcome to bone health, the use of micronutrient supplementation should be considered [35]. In a recent study of bone mineral density conducted in Europe, adult PKU participants had density studies within normal ranges [39]. This suggests that in depth bone health assessments could focus on patients who are at higher risk for fractures, hormone imbalance, alcohol abuse or other factors.

### New treatments

#### Sapropterin

The ACMG and European guidelines recommend the use of sapropterin in patients with PAH deficiency [3, 6]. Their approaches to testing the drug for patient responsiveness differed in length of time to trial the medication and whether to continue it if there is not concomitant drop in phenylalanine levels. At the time of the publication of the European guidelines, there was limited research evidence, but more patient use information available about the medication. Use of sapropterin during pregnancy was noted in the ACMG guidelines [3]. To reiterate, patients already on sapropterin could be counseling about the known effects of the medication on the fetus. Pregnant women with high phenylalanine levels could start the medication to lower phenylalanine levels and avoid teratogenic effects to the fetus.

Adherence to sapropterin was associated with increased allowances of dietary protein [40, 41]. A 2017 European study established sapropterin as safe for use in children younger than age four [42]. Adherence to oral sapropterin was explored; patients were more likely to continue taking the medication if it allowed for increased protein intake [40]. Side effects and insurance issues were major reasons for discontinuing the medication. That sapropterin did not work as well as expect was also cited as a cause for stopping the medication. Successful outcomes cases for maternal phenylketonuria and sapropterin treatment were noted in several studies [43, 44]. An international team of researchers developed guidelines for testing and establishing responsiveness to sapropterin [45].

#### Pegvaliase

Pegvaliase is an injectable PAH deficiency treatment which was approved for patient use in the U.S. in 2019. When the original guidelines were written, pegvaliase was not yet approved and there was limited information about it in the guidelines. Since that time, a number of studies have been conducted to evaluate its efficacy and safety. Pegvaliase acts through an alternative enzymatic pathway to metabolize phenylalanine. Pegvaliase converts phenylalanine into trans-cinnamic acid and ammonia, which is excreted in urine. Early research shows pegvaliase effectively lowering phenylalanine levels, enabling individuals to increase the natural protein in their diets [46]. However, there are reports of hypersensitivity reactions and side effects related to the medication [47]. The most severe reaction being anaphylaxis. Other reported adverse events include injection site erythema, arthralgia, and headache among others [47]. Immune system activation can be expected, but lessening of this may occur over time [48, 49]. Many clinical trial patients achieved sustained reductions in phenylalanine levels [47] Pegvaliase appears to be effective in lowering phenylalanine levels for people regardless of with PAH deficiency phenotypes [46]. Longo et al. developed recommendations for treating adults with pegvaliase, and there is a need for long-term research on the safety, dosing, and management of patients using pegvaliase in this newly approved treatment [50].

## Discussion

The ACMG and European guidelines revealed many similarities and some differences in their respective approaches to PAH deficiency diagnosis, treatment, and management.

There were differences in the choosing the composition of personnel for the respective ACMG and European teams for developing the guidelines. The ACMG group members were self-referred to participate in developing the guidelines and conflicts of interest of committee members were registered with the ACMG. In a commentary on the European work group, it was suggested that fewer countries were represented in the group membership [51, 52]. Group composition may influence guideline conclusions and recommendations [53]. Group members should have relevant clinical and research expertise, but also represent diverse stakeholders including patients and families, researchers, clinicians, advocacy groups and others.

Both the ACMG and key European work groups encountered difficulties identifying strong, evidence-based studies to support their recommendations for PAH deficiency care. At the time of publication of the guidelines, there was limited information and evidence available in some areas, particularly in new treatments, but this has increased greatly with more studies produced in the intervening years.

Newborn screening is necessary for the early detection of PAH deficiency as noted in both guidelines. The types of accompanying diagnostic testing were varied, but differentiating BH_4_ defects from PAH deficiency was noted by both groups. Genotyping was recommended by the ACMG. The European group favored genotyping for determining BH_4_ responsiveness. Garbade, et al. suggested that genotype/phenotype predictions for PAH deficiency may be enhanced by using large data sets to define allelic phenotype/ genotype phenylalanine values and pretreatment sapropterin responsiveness [54]. Li et al. supported PAH deficiency genotyping as a factor in phenotypic presentation in a study of mainland Chinese population [11].

The therapeutic ranges for phenylalanine levels differ in the guidelines. The ACMG recommends diet for life with phenylalanine levels maintained between 120 and 360 μmol/L. The European guidelines recommend treatment for phenylalanine levels > 360–600 μmol/L in children younger than age 12. For teens and adults treatment should be continued for levels > 600 μmol/L. It was suggested that dietary restrictions to maintain these phenylalanine levels may be too stringent and difficult to maintain and higher phenylalanine levels may not lead to psychological difficulties in every case [51]. Other evidence suggests that the 360 μmol/L target for phenylalanine levels may be too high and neurocognitive benefit may be observed by lowering this value [23].

The schedules for ongoing phenylalanine monitoring are the same for both the ACMG and European guidelines. There are differences in frequencies and types of nutritional testing and biochemical assessments performed for persons with PAH deficiency, though both groups agree that increased testing is warranted if clinically indicated. Both guidelines recommend that clinical care be conducted using multidisciplinary teams. Scheduling of clinic visits was comparable between the guidelines with more frequent visits for infants and pregnant women.

The ACMG and European experts view treatment adherence as a very serious concern. Exploring ways to improve dietary compliance is an area in need of research with the creation of appropriate, effective interventions [24]. Treatment adherence rests on time management and organizational skills to ensure good compliance, but there are challenges to maintaining the low protein diet, daily metabolic formula intake, and monitoring phenylalanine levels. High phenylalanine levels can affect executive function and organizational abilities, which impairs decision- making, treatment planning, and tasks such as meal preparation [3, 5]. Children with PAH deficiency do well with treatment while in the care of their parents, but treatment adherence lessens with age [4, 5].

The development of new treatments, such as sapropterin and pegvaliase, can help to lower phenylalanine levels through means other than diet and may improve adherence and maximize patient outcomes. These treatments are not curative, but offer patients and families the potential for better phenylalanine control.

## Conclusions and recommendations

PAH deficiency is one of the earliest identified inborn errors of metabolism amendable to treatment. Research has greatly enhanced provider knowledge related to PAH deficiency treatment and management. Enhanced evidence-based practice is crucial for optimizing health, improving quality of life, and neurocognitive outcomes of individuals with PAH deficiency. Both the ACMG and key European guidelines agree there is a need for stronger evidence for improving care of persons with PAH deficiency.

In forming workgroups for guideline development, diverse stakeholders represent a variety of interests, but it is important that those engaged be as free as possible from potential bias stemming from commercial or research interests. A methodologist skilled in creating systematic reviews and meta-analyses could be added to the work groups to help in evaluating and organizing the research evidence [53].

Neurocognitive assessments frequency of testing and the types of instruments used for screening differed between the guidelines. Neurocognitive and behavioral issues can be associated with phenylalanine hydroxylase deficiency, especially when phenylalanine levels are elevated. Careful attention to phenylalanine levels and application of neurocognitive evaluations are important to intervene appropriately with therapies and educational support when needed.

There is controversy for what constitutes optimal phenylalanine levels for young persons and adults. The ACMG recommendations for phenylalanine levels are invariant across the lifespan, while the European guidelines afford different cut offs according to age. Perhaps in the future the question of phe levels will be agreed upon and fully clarified. For now, treatment adherence can pose challenges to patients, families, and clinicians. Treatment cannot be so onerous that it is very difficult to follow. At the same time, it must be strict enough to protect people from the adverse consequences of high phenylalanine levels. Treatment adherence also affects quality of life. The choices that patients and their families make are based on their best judgments with guidance from clinicians. Ideally, treatment should be an integral part of daily routines, and not cause guilt or untoward hardship when it is not sustained. An approach that includes attention to individual education and skills building, incorporation of new developments in diet, formulas and other treatments when appropriate, and supporting and closely working with patients and families, can help in identifying and reaching the desired goals. Research can help identify factors that support patient progress in their care.

This comparison paper attempted to provide a summary of U.S. and European guidelines for the management of PAH deficiency. We tried to account for recommendations within the clinical guidelines, but recognize there is a chance that we may have missed some of these or insufficiently highlighted others. As updates and refinements of guidelines in the coming years are considered, systematic reviews of new evidence would be a good start to guideline review. The ACMG and European group reached similar conclusions for many aspects of care of patients. Perhaps now is the time for organizations to consider the development of world-wide committees to examine evidence related to PAH deficiency care.

Expert clinical care and research leading to new developments in treatment are testaments to researchers and clinicians who through continual work and dedication try to make life better for those affected by PAH deficiency. However, without patient and family collaboration, research would be impossible. It is through the combined efforts and hopes of patients, parents, families, clinicians, and researchers that appreciable gains can be made in the lives of persons with PAH deficiency.

## Supplementary information

**Additional file 1: Supplement 1.** Listing of Guideline Topics by Page Number in Isolated PDF Versions.

## Data Availability

The articles included in this review are publicly available.

## Abbreviations

PAH: phenylalanine hydroxylase
ACMG: American College of Medical Genetics and Genomics
ESPKU: European Society for Phenylketonuria and Allied Disorders
PKU: Phenylketonuria
NIH: National Institutes of Health
AHRQ: Agency for Healthcare Research and Quality
SIGN: Scottish Intercollegiate Guideline Network
AGREE: Appraisal of Guidelines for Research and Evaluation
GMDI: Genetic Metabolic Dieticians International
MPKU: Maternal PKU
GMP: Glycomacropeptide
LNAA: Large neutral amino acids
PEG-PAL: Polyethylene-glycol conjugated phenylalanine ammonium lyase
QoL: Quality of life
