# Supplementary material for: Similarities and differences in key diagnosis, treatment, and management approaches for PAH deficiency in the United States and Europe
Source: Orphanet J Rare Dis. 2020 Sep 25;15:266. doi: 10.1186/s13023-020-01541-2 (PMC7519570; doi:10.1186/s13023-020-01541-2)
Supplement: Supplementary file 1 — Additional file 1: Supplement 1. Listing of Guideline Topics by Page Number in Isolated PDF Versions. [file 13023_2020_1541_MOESM1_ESM.docx]

| **Supplement 1. Listing of Guideline Topics by Page Number in Isolated PDF Versions** | | | |
| --- | --- | --- | --- |
| **Topic**  Methods  Screening and Diagnosis  Treatment Initiation  Treatment Duration  Female Specific Treatment Recommendations  Routine Monitoring  Dietary Treatment  Pharmacological Treatment  Emerging Therapies  Maternal PKU  Late or Untreated PAH Deficiency  Genetic Counseling | **Vockley et al. (2014)** | **van Spronsen et al. (2017)** | **van Wegberg et al. (2017)** |
|  | p. 2  pp. 2-3  pp. 3-4  p. 6  p. 7  p. 5  p. 4  pp. 5-6  p. 6  pp. 7-8  pp. 6-7  p. 8 | p. 2  p. 3  p. 3  pp. 3-4  p. 4  pp. 9-10 (table 2)  pp. 7-8  p. 8  N/A  pp. 10-11    p. 11  N/A | pp. 3-4    pp. 5-6  p. 7  pp. 7-8  pp. 30  pp. 14-15 (table 2)  pp. 20-29  pp. 41-42  p. 42  pp. 30-38  p. 39  p. 6 |
